# Smoking Patterns of University Woman Students in Miyagi, Japan: The Miyagaku Study

**DOI:** 10.2188/jea.13.296

**Published:** 2007-11-30

**Authors:** Hiroko Iwaoka Ohwada, Takeo Nakayama

**Affiliations:** 1 Department of Food and Nutritional Science, Miyagi Gakuin Women’s University.; 2 Department of Medical System Informatics, School of Public Health, Kyoto University.

**Keywords:** smoking, students, Japanese women, cross-sectional studies

## Abstract

BACKGROUND: The increase in the smoking rate among young women is a worldwide problem. However, few reports have focused on female students, in particular, with detailed accounts of their smoking behavior. The aim of this study was to clarify the smoking patterns of Japanese women of approximately 20 years of age.

METHODS: Smoking behavior, age at initiation, favorite brand and related attitudes were examined using a cross-sectional anonymous questionnaire administered to students at a women’s university in Miyagi, Japan in 2000.

RESULTS: Of 2,984 subjects (response rate: 96%), 16% said that they smoke (95% confidence interval 15-18%): 7% of freshmen, 16% of sophomores, 22% of juniors and 20% of seniors. While music majors were most likely to smoke (21%), domestic science majors had the lowest rate of smoking (10%). Among the smokers, 27% started the habit at age 20 years, the legal age in Japan, and 25% started at age 18. The favorite brand was Marlboro (39%), followed by Mild Seven (16%), a domestic brand. One-third of the smokers had no plans to quit.

CONCLUSIONS: Reaching the legal age and entering university may prompt young women to start smoking habitually. In contrast to its overall market share in Japan, a US brand is now favored by current young female smokers.

The annual number of smoking-related deaths worldwide has been estimated at five million. The World Health Organization has officially announced that anti-smoking measures are among its major priorities.^1^^,^^2^ Many experts have emphasized the need to focus on anti-smoking measures for young people, including minors, and women. The methodology used for such measures should be different from the conventional approach, in which most smokers are assumed to be adult males.

When discussing youth smoking, the focus has been on under-aged students in elementary, junior high, and senior high school.^3^ Findings regarding university students, who are on the borderline between minors and adults, are relatively uncommon. Some previous reports have examined male students only,^4^^-^^6^ or the analyses were conducted irrespective of sex.^7^^-^^10^ Studies have focused on the age at which they started smoking,^4^^,^^10^^-^^15^ smoking rate,^8^^,^^10^^,^^11^^,^^13^^,^^15^^-^^18^ the number of cigarettes smoked per day,^12^^,^^14^^,^^17^^-^^19^ smoking frequency,^9^^-^^11^ the reasons for starting to smoke^12^^,^^15^ or for not smoking,^10^ opinions regarding smoking cessation,^10^^,^^12^^,^^13^^,^^15^ behaviors related to smoking habits,^10^^,^^19^^,^^20^ environmental factors such as smoking habits of family members,^4^^,^^10^^,^^12^^,^^15^^,^^18^ and comparison of serum elements including beta-carotene in smokers and nonsmokers.^5^ However, there have been few reports focusing on smoking among female students and their awareness of the risks of smoking.

The smoking rate in Japan has been characterized as very high in men (50-60%) and low in women (10-15%). However, according to the results of a survey by Japan Tobacco Inc., smoking among women in their 20s of age has increased markedly in recent years from 7% in 1965 to 22% in 2000 (http://www.health-net.or.jp/tobacco/product/pd090000.html). Information specified to smoking behaviors in women around the age of 20 years is needed because this is the legal smoking age in Japan. Such information, however, is lacking both in Japan and elsewhere around the world. 

As a part of the project called the Miyagaku Study, we conducted a pilot study in 1999 to collect basic information regarding the smoking behaviors of female university students. In 2000, the first cross-sectional study was conducted to analyze these students’ smoking behaviors and related factors. This article presents descriptive information from the results of this cross-sectional study.

## METHODS

Sendai City is the largest city in the Tohoku District and the 11th largest city in Japan, with a population of one million as of October 2000. Miyagi Gakuin Women’s University, where this study was conducted, is located in Sendai City in Miyagi Prefecture, about 20 km from the central part of Sendai City. Ninety percent of the students are from the 6 prefectures of the Tohoku District. The study surveyed students majoring in English or Japanese literature, social sciences, home economics, food and nutritional science, and music. The students in English and Japanese literature, and social sciences were categorized as literature majors; those in home economics, and food and nutritional science as domestic science majors; and those in music as music majors.

As shown in Figure, in April 2000, the beginning of the new school year in Japan, the college had a total enrollment of 3,248, composed of 33 postgraduates, 2,842 undergraduates, and 373 junior college students. We distributed questionnaires to 3,118 students during their medical checkups at the beginning of the school year. Because the survey was conducted at an academic institution, and minors, who are legally prohibited from smoking in Japan, were included among the subjects, the survey was conducted anonymously. The questionnaires were distributed to the students at the medical checkup site and were filled out on the spot. The survey staff checked for blank items when collecting the questionnaires from the students. While 134 did not respond to the questionnaire, 96% or 2,984 students responded. For this analysis, which particularly focused on university undergraduates across the four years of their schooling, we excluded 701 respondents, namely, 22 postgraduates, 356 junior college students, and 323 university students from departments in which students of all classes (freshmen, sophomores, juniors, and seniors) were not available. This left 2,283 students, which was equal to 80% of the total number of the students in this college. A total of 1,683 of these subjects were literature majors (628 were studying social sciences; 574, Japanese literature; 481, English literature), 412 were domestic science (nutrition) majors, and 188 were music majors.

**Figure.**
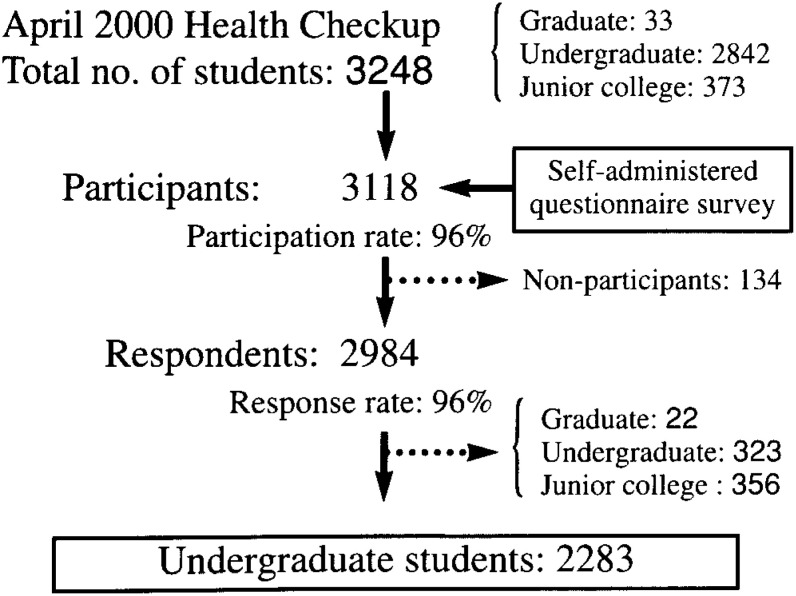
Sampling frame of the Miyagaku Study, Sendai, Japan, 2000.

The survey focused on the students’ smoking behaviors, the degree of understanding of smoking-associated risks, the Fagerström test for nicotine dependence,^21^ Prochaska’s model for the stages of change,^22^ modeling based on social learning theory,^23^ self-efficacy of quitting smoking, the perceived influence of the mass media, the association between diet or weight control and smoking behaviors, and the perception of mild (low-yield) cigarettes.

Respondents were first asked to report whether they smoked. Subsequently, one of the questions asked the respondents how many days they had smoked during the past 30 days. Respondents with answers ranging from 1 to 30 days were regarded as current smokers. This question had been used previously in a nation-wide survey of junior and senior high school students, and was thought to generally be answered candidly by minors.^3^ We used this item because minors were included in our subjects, and also because the same questions had to be used for all students, from freshmen, many of whom were minors to seniors. This survey reveals many aspects relating to smoking patterns among female university students, and focuses on: (1) the smoking rate particularly among female students, (2) the age at which they started smoking, (3) the brands of cigarettes they smoke, and (4) their attitudes towards smoking.

We used the Mantel extension method to test whether the smoking rate tended to be higher among seniors. Fisher’s exact tests or the Mantel-Haenszel method for pair comparison and Bonferroni’s test for multiple comparisons were used. The 95% confidence interval (CI) was calculated from the following equation:

$$\frac{n}{n+Z^{2}}\left(p+\frac{Z^{2}}{2n}\pm Z\sqrt{\frac{p(1-p)}{n}+\frac{Z^{2}}{4n^{2}}}\right)$$

where Z is 1.96, n is the sample size, and p is the proportion. SPSS^®^ Version 10 was used for statistical analysis with a significance level of 5% or lower.

## RESULTS

The smoking rates for students of different classes were as follows: 7% for freshmen, 16% for sophomores, 22% for juniors, and 20% for seniors. As shown in Table 1, the overall smoking rate was 16%. The smoking rates increased with the year of study, except that the smoking rate among seniors was slightly lower than that of juniors. This tendency remained even when students were categorized according to their majors using the indirect method. The smoking rate among seniors turned out to be slightly lower than that of juniors in domestic science, significantly lower in music (p<0.05), but slightly higher in literature. The overall smoking rate for each major was as follows: 10% in domestic science, 17% in literature, and 21% in music. The smoking rates among literature and music majors were significantly higher compared to that among domestic science majors (p=0.001 and p<0.001, respectively). This tendency remained even when their classes were adjusted using the indirect method.

**Table 1.**
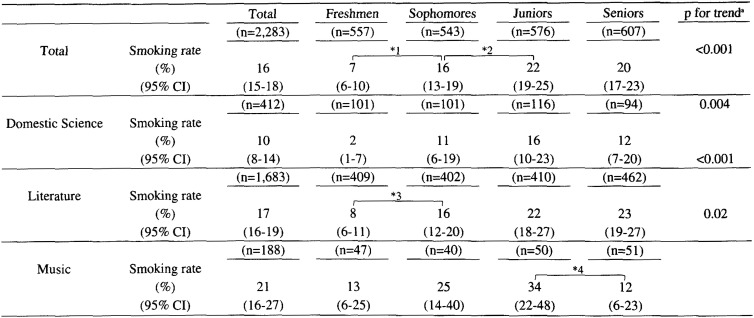
Smoking rate among university women according to major: The Miyagaku Study, Sendai, Japan, 2000.

CI: Confidence Interval.

^a^ Mantel Extention Method.

*1-*4: Bonferroni’s multiple comparison method. Fisher’s exact method is used for paired-comparison.

*1: p<0.001, *2: p=0.007, *3 p<0.001, *4: p=0.009

Of all the students who smoked, 27% indicated that they started smoking when they were 20 years of age, followed by 25% who started smoking at the age of 18 (Table 2). Among the student smokers 20 years old and older, 39% of the juniors (3rd year) and 42% of the seniors (4th year) started smoking when they were 20. Twenty-two percent of the smokers aged 20 or older reported that they started smoking when they were 18 years old.

**Table 2.** Age at which students started smoking: The Miyagaku Study, Sendai, Japan, 2000.

Age(year)	Total (n=277) ^a^	Freshmen (n=29)	Sophomores (n=62)	Juniors (n=97)	Seniors (n=89)
13	1 (0)			1 (1)	
14	9 (3)	5 (17)	1 (2)	1 (1)	2 (2)
15	13 (5)	5 (17)	6 (10)	2 (2)	
16	24 (9)	8 (28)	7 (11)	7 (7)	2 (2)
17	23 (8)	6 (21)	10 (16)	5 (5)	2 (2)
18	69 (25)	5 (17)	24 (39)	19 (20)	21 (24)
19	55 (20)		13 (21)	23 (24)	19 (21)
20	76 (27)		1 (2)	38 (39)	37 (42)
21	7 (3)			1 (1)	6 (7)

The denominators of the proportions are self-reported smokers of each major and grade with eligible responses.

^a^ Seven self-reported smokers did not answer the age at which they started smoking.

Percentages in parentheses.

With regard to the brands, 39% the students chose Marlboro as their favorite brand, followed by 16% who chose Mild Seven (Table 3). Marlboro was chosen as the favorite brand among all majors and in each class. Mild Seven was the second-favorite brand among freshmen, sophomores, and juniors, but Virginia Slims was the second-favorite among seniors. This trend existed regardless of the age at which they started smoking. Marlboro was considered the favorite brand by 37% of the students who started smoking between the ages of 13 and 17, by 42% of those who began smoking at the age of 18-19, and by 36% of those who started smoking at the age of 20-21.

**Table 3.** Favorite cigarette brands of university women: The Miyagaku Study, Sendai, Japan, 2000.

	Total (n=284)		Freshmen (n=31)		Sophomores (n=64)		Juniors (n=100)		Seniors (n=89)	
				
	Brand	n (%)	Brand	n (%)	Brand	n (%)	Brand	n (%)	Brand	n (%)
1	Marlboro	112 (39)	Marlboro	16 (52)	Marlboro	24 (38)	Marlboro	34 (34)	Marlboro	38 (43)
2	Mild Seven	44 (16)	Mild Seven	4 (13)	Mild Seven	13 (20)	Mild Seven	17 (17)	Virginia Slims	13 (15)
3	Virginia Slims	36 (13)	Virginia Slims	3 (10)	Virginia Slims	7 (11)	Virginia Slims	13 (13)	Mild Seven	10 (11)
4	Salem	27 (10)	Lucky Strike	3 (10)	Salem	5 (8)	Salem	12 (12)	Salem	10 (11)
5	Kool	16 (6)	Others	5 (16)	Frontier	3 (5)	Kool	7 (7)	Kool	6 (7)
6	Lucky Strike	5 (2)			Others	12 (19)	Philip Morris	3 (3)	Others	12 (14)
7	Frontier	4 (1)					Others	14 (14)		
8	Philip Morris	3 (1)								
9	Others	27 (10)								

The denominators of the percentages are self-reported smokers of each major and grade with eligible responses.

As shown in Table 4. 41% of the students who smoked answered that they wanted to cut down on their smoking when asked whether they were considering about quitting smoking, while 30% responded that they wanted to quit smoking. When asked whether they were able to quit smoking, 36% of the respondents answered that they were able to quit within a year, and another 36% answered that they could quit if they became pregnant. When asked if they were interested in quitting smoking, 56% answered that they were interested in quitting but that they were not going to quit within a month. Furthermore, according to the age at which they started smoking, we studied the students’ intention, self-efficacy, and concern to quit smoking. The highest percentage of students who answered affirmatively to the statements: “I have no intention of quitting smoking (7%)”; “I will probably not be able to stop smoking (12%)”; and “I am not concerned in smoking cessation (28%)”, was registered in the group of students who started smoking between the age of 13-17 years in contrast to those who started smoking at the age of 18-19 and 20-21.

**Table 4.** Smokers’ attitudes regarding smoking cessation: The Miyagaku Study, Sendai, Japan, 2000.

Intention	Total	Really want to quit		Want to cut down		Kind of want to quit		Never want to quit	
					
Do you want to quit smoking?	n	n	%	n	%	n	%	n	%

	284 ^a^	84	30 (24-35)	115	41 (35-46)	69	24 (19-29)	16	6 (3-8)
					
Self-efficacy	Total	Can quit within a year		Can quit if I become pregnant		Can quit, but don’t know when		Cannot quit	
					
Can you quit smoking?	n	n	%	n	%	n	%	n	%
	
	284 ^a^	102	36 (30-42)	102	36 (30-42)	57	20 (16-25)	22	8 (5-11)
					
Concern	Total	Want to quit smoking as soon as possible		Concerned, but have no plan to quit smoking within a month		Not concerned			
				
Are you concernced about quitting smoking?	n	n	%	n	%	n	%		

	284 ^a^	73	26 (21-31)	159	56 (50-62)	52	18 (14-23)		

The denominators of the percentages are self-reported smokers of each major and grade with eligible responses.

^a^ : Among 372 current smokers, they had smoked on one or more days in the previous 30 days, 284 persons reported themselves as regular smoker.

## DISCUSSION

The percentages of smokers among university students have elsewhere been reported to be 22-48% for men and 7-37% for women.^24^^-^^28^ However, only the limited number of reports from Asia, including Japan, have been published, and few are available in English.

The response rate of the present study was 96%. Such a high response rate was attributed to the fact that the questionnaires were distributed during one of the regular medical checkups held at the university. Thus, all students who had come to receive a medical checkup were asked to participate in the survey. If the questionnaire survey had been conducted in class, students might have felt undue pressure to participate, thinking that their refusal to participate might influence their grades. The present survey was therefore conducted outside the class. Students were asked to participate in the survey during a medical checkup and their participation was voluntary. Nevertheless, it is possible that students felt that they were under some sort of pressure. In addition, at the time of the survey, April 2000, the governmental ethical guidelines for research, mandating anonymous self-administered questionnaires, were not available. In order to minimize undue pressure, informed consent documents for more recent surveys include a statement according to which subjects are not forced to answer those questions they do not wish to answer.

In the present study, respondents who had smoked on one or more days in the previous 30 days were considered as current smokers. This method was adopted in a nation-wide survey of elementary and junior high school students in Japan.^3^ Minors who are hesitant to report their smoking habit might answer this question relatively easily. However, the results from this method were somewhat different from those of self-reported regular smoking. Based on the question described above and previously used to survey minors, the overall smoking rate at the university used in the current study was calculated to be 16%. However, among the respondents, 12% answered the question regarding the age at which they started smoking habitually, and these students were classified as considering themselves regular smokers. The university is a traditional one, and the most students are from upper-middle social class. Therefore, the smoking rate may not be as high as it is at other women’s universities or among working women of the same age group. When asked how many days they had smoked in the previous 30 days, six students answered zero days while at the same time responding to the question regarding the age at which their smoking became habitual. Likewise, 10 students said that they had smoked on one to two days while also responding to the question regarding when their smoking became habitual. The largest number of respondents, 157 students, stated that they had smoked every day during the previous 30 days and responded to the question regarding when their smoking became habitual. By adding this number to the 46 students who answered that they had smoked on 20 to 29 days during the previous 30 days, it was determined that 203 students, or 72% of all respondents who smoked, considered themselves habitual smokers. The definition of “habitual” in the question regarding the age at which students became habitual smokers was unclear, but the results of this survey indicated that most respondents considered smoking 20 or more days during the previous 30 days as habitual. The percentage of respondents who smoked everyday was 7%. According to the 1998 Survey on Smoking and Health,^29^ the smoking rate among 15 to 19-year-old individuals was 19% for males and 4% for females. The present survey included 1,054 under-aged respondents (18-19 years old) and the smoking rate among them was 11%. The smoking rate reported in the present survey was higher than that in the 1998 Survey on Smoking and Health, perhaps because the respondents participating in the present study were older than those participating in the 1998 survey.

The smoking rate was low among domestic science majors, and high among literature and music majors. In those majors with high smoking rates, the students had started smoking at a relatively early age, e.g. at age 14 or 15 years. Metintas et al. in Turkey^16^ and Bard et al. in Hong Kong^30^ have analyzed smoking rates among university students by major. According to Metintas, the highest smoking rate, 61%, was found among arts majors. This was followed by 50% among education majors and 41% among engineering majors. The lowest smoking rate, 34%, was found among medical majors. Bard reported that the highest smoking rate among students of all majors both at the time of and after entry into the university was among male students majoring in social sciences (6% at the time of and 26% after entry into the university). In the present survey, the lowest smoking rate was detected among students who majored in domestic science. Respondents from domestic science majors comprised only those who participated in a nutrition training course, of which curriculum required that students study health-related subjects such as public health, clinical medicine, nutrition guidance, and health promotion and management. In other words, one difference between these students and those from other majors lies in that their curriculum gave them access to information about the effects of smoking on health. In fact, the highest smoking rate was registered among students of music, of which curriculum included no subjects about health. This result suggests that in order to reduce smoking among university students, it may be necessary to regularly provide them with opportunities to learn how smoking affects health.

According to a nation-wide survey in Japan, the current rates of smoking among third year high school students, who were approximately 18 years old, were 26% for men and 5% for women.^3^ The present findings imply that the smoking rate among women may increase sharply after graduation from high schools. In Japan, reaching the legal age of 20 years and entering a university may prompt women to start smoking. When entering university, they encounter many older smokers, and cigarettes become more available.

Types of cigarettes, such as “light,” “super light,” or “menthol,” were not specified in the questionnaire; we focused only on the brands. The top-ranking brands among all majors were foreign brands, such as Marlboro, and Mild Seven was the only domestic brand that was classified as a favorite. These results indicate that female students favor foreign brands. This trend remained regardless of the age at which they started smoking. Marlboro was found to be the most popular brand for every age at which they began smoking.

A survey of students in 12 universities in China showed that Marlboro was very popular.^31^ Four of the top eight brands were foreign: Marlboro, 555, Kent, and Hilton. Advertisements for the foreign brands were much more likely to be seen than those for domestic brands; advertisements for Marlboro were reported most often (30%), followed by those for 555 (22%) and Kent (18%). Among smokers, Marlboro was the most popular foreign brand, preferred by 44%. Our findings also reflected Marlboro’s high recognition rate among young people in Asia. In the Japanese tobacco market, Mild Seven has the top market share of 29% and Marlboro 8% (http://www.jti.co.jp/JTI/tobacco/data/data4.html). The present survey revealed that Marlboro was the favorite brand among female university students. In addition, while Marlboro has been considered a masculine brand for a long time, their manufacturer is aiming at expanding their market by making their advertisements more appealing to female smokers. Marketing strategies of tobacco manufacturers are important factors that influence the smoking rate among female students. The reason why students chose Marlboro as their favorite brand has not been identified in the present survey. Therefore, a subsequent ongoing survey includes an item asking students why they chose a particular brand.

The percentages of respondents who answered, “I really want to quit” was 38% among music majors, with the highest smoking rate, followed by 29% among literature majors, with the second-highest smoking rate, and 28% among domestic science majors, with the lowest smoking rate. The total percentages of students who wanted to quit and those who wanted to cut down on smoking were 76% for music majors, 70% for literature majors, and 66% for domestic science majors. However, the fact that one-third of smokers among the university women persist in their smoking habits can be considered serious. The percentages of smokers who wanted to quit or cut down on smoking were higher in the departments with higher smoking rates. The reason is unclear; however, even students in majors where smoking rates are high may be receptive to programs for smoking cessation. In the 17 years of age or younger smoking opener, the students’ intention, self-efficacy, and concern to quit smoking were lower than the 18 or older-year-old smoking opener. Although an objective of this study was to provide a basis for developing a smoking cessation program for female university students, the results also suggest the importance of development of the smoking cessation program in younger population.

We conclude that reaching the legal age and entering university may prompt young women to start smoking habitually. Considering its overall market share in Japan, a US brand is disproportionately popular among young women who are just beginning to smoke. Related behaviors and attitudes need to be further studied to develop an effective intervention. Despite its importance, information about smoking behaviors among female university students is seriously lacking. Integration of an environmental approach, such as smoking restrictions in public areas, and developing smoking cessation programs focusing on individuals may be useful university-based anti-tobacco measures. More information must be obtained concerning the characteristics of subjects and their community.
